# The Practice of Deep Sedation in Electrophysiology and Cardiac Pacing Laboratories: Results of an Italian Survey Promoted by the AIAC (Italian Association of Arrhythmology and Cardiac Pacing)

**DOI:** 10.3390/jcm10215035

**Published:** 2021-10-28

**Authors:** Pietro Palmisano, Matteo Ziacchi, Andrea Angeletti, Federico Guerra, Giovanni Battista Forleo, Matteo Bertini, Pasquale Notarstefano, Michele Accogli, Carlo Lavalle, Giovanni Bisignani, Maurizio Landolina, Gabriele Zanotto, Antonio D’Onofrio, Renato Pietro Ricci, Roberto De Ponti, Giuseppe Boriani

**Affiliations:** 1 Cardiology Unit, “Card. G. Panico” Hospital, 73039 Tricase, Italy; accogli.michele@libero.it; 2 Institute of Cardiology, S. Orsola-Malpighi University Hospital, University of Bologna, 40138 Bologna, Italy; matteo.ziacchi@gmail.com (M.Z.); andrea.angeletti89@gmail.com (A.A.); 3 Cardiology and Arrhythmology Clinic, Marche Polytechnic University, University Hospital Umberto I-Lancisi-Salesi, 60126 Ancona, Italy; guerra.fede@gmail.com; 4 Department of Cardiology, Ospedale Luigi Sacco, 20157 Milano, Italy; forleo@me.com; 5 Cardiology Unit, Azienda Ospedaliero-Universitaria Di Ferrara “Arcispedale S. Anna”, 44124 Cona, Ferrara, Italy; doc.matber@gmail.com; 6 Cardiovascular Department, San Donato Hospital, 52100 Arezzo, Italy; pasqualenotarstefano@gmail.com; 7 Department of Cardiology, Policlinico Universitario Umberto I, 00161 Roma, Italy; carlolavalle@yahoo.it; 8 Cardiology Division, Castrovillari Hospital, ASP Cosenza, 87012 Castrovillari, Italy; giovanni.bisignani@virgilio.it; 9 Cardiology Department, Ospedale Maggiore Di Crema, 26013 Crema, Italy; maurizio.landolina02@gmail.com; 10 Department of Cardiology, Mater Salutis Hospital, 37045 Legnago, Verona, Italy; gabriele.zanotto62@gmail.com; 11 Departmental Unit of Electrophysiology, Evaluation and Treatment of Arrhythmias, Monaldi Hospital, 80131 Naples, Italy; donofrioant1@gmail.com; 12 Centro Cardio-Aritmologico, 00152 Roma, Italy; renatopietroricci@gmail.com; 13 Department of Heart and Vessels, Ospedale Di Circolo-University of Insubria, 21100 Varese, Italy; roberto.deponti@uninsubria.it; 14 Department of Biomedical, Metabolic and Neural Sciences, Cardiology Division, University of Modena and Reggio Emilia, Policlinico Di Modena, 41121 Modena, Italy; giuseppe.boriani@unimore.it

**Keywords:** ablation, cardioversion, electrophysiology, implantable cardioverter defibrillator, pacemaker, sedation

## Abstract

The aim of this survey, which was open to all Italian cardiologists involved in arrhythmia, was to assess common practice regarding sedation and analgesia in interventional electrophysiology procedures in Italy. The survey consisted of 28 questions regarding the approach to sedation used for elective direct-current cardioversion (DCC), subcutaneous implantable cardioverter-defibrillator (S-ICD) implantation, atrial fibrillation (AF) ablation, ventricular tachycardia (VT) ablation, and transvenous lead extraction procedures. A total of 105 cardiologists from 92 Italian centres took part in the survey. The rate of centres where DCC, S-ICD implantation, AF ablation, VT ablation and lead extraction procedures were performed without anaesthesiologic assistance was 60.9%, 23.6%, 51.2%, 37.3%, and 66.7%, respectively. When these procedures were performed without anaesthesiologic assistance, the drugs (in addition to local anaesthetics) commonly administered were benzodiazepines (from 64.3% to 79.6%), opioids (from 74.4% to 88.1%), and general anaesthetics (from 7.1% to 30.4%). Twenty-three (21.9%) of the 105 cardiologists declared that they routinely administered propofol, without the supervision of an anaesthesiologist, in at least one of the above-mentioned procedures. In current Italian clinical practice, there is a lack of uniformity in the sedation/analgesia approach used in interventional electrophysiology procedures.

## 1. Introduction

The number of complex electrophysiological procedures has increased over recent decades. These procedures can be painful and immobility of the patient is necessary for the success of the procedure. For this reason, deep sedation or general anaesthesia are frequently used. While the former can be achieved through a proceduralist-directed nurse-administered (PDNA) approach, the latter requires anesthesiologic support. The PDNA approach has the advantage of eliminating the need for anaesthesiologists and can reduce pre-procedure time. On the other hand, safety warnings on deep sedation are reported in the literature, particularly regarding the need to switch to general anaesthesia or to undertake urgent airway interventions [1,2].

The American Society of Anesthesiologists (ASA) and the American College of Emergency Physicians (ACEP) have provided guidelines concerning sedation/analgesia [3,4]. Strong evidence regarding the optimal pharmacological protocol is lacking and often equivocal.

This Italian survey, endorsed by the Italian Association of Arrhythmology and Cardiac Pacing (AIAC), aimed to evaluate common practice regarding sedation and analgesia in electrophysiology laboratories (EP labs) in Italy.

## 2. Materials and Methods

From 1 March to 15 December 2020 a survey endorsed by the AIAC was conducted on the official AIAC website (https://aiac.it/, accessed on 15 April 2021). The survey was open to all Italian cardiologists involved in arrhythmia care, and participation was voluntary. The questionnaire could be completed by more than one cardiologist from the same centre and was composed of 28 questions: 13 on the characteristics of the hospital (i.e., number of beds; availability of a surgery, anaesthesiology and intensive care unit (ICU); types and annual volume of interventional electrophysiology procedures); 15 concerned the approach to sedation used for elective direct-current cardioversion (DCC), subcutaneous implantable cardioverter-defibrillator (S-ICD) implantation, atrial fibrillation (AF) ablation, ventricular tachycardia (VT) ablation, and transvenous lead extraction procedures. Twenty of the 28 questions were multiple-choice questions (see online Supplementary Materials for Details). 

### Statistical Analysis 

Descriptive statistics were reported as means and standard deviations for normally distributed continuous variables. Continuous variables with skewed distribution were reported as medians with 25–75th percentiles. Categorical data were expressed as percentages, reported in contingency tables, and compared by means of *χ*^2^ test or Fisher’s exact test, as appropriate. Odds ratios (ORs) were reported with their 95% confident intervals (CIs) and *p* values < 0.05 were considered as statistically significant. The data were analysed using the statistical software package Statistica version 6.1 (StatSoft Inc., Tulsa, OK, USA).

## 3. Results

### 3.1. Participating Centres

**Figure 1:**
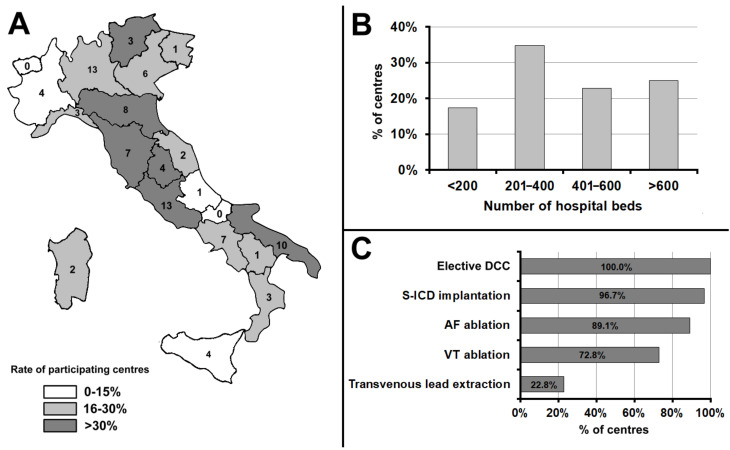
(**Panel A**): Geographic distribution of the centres that responded to the survey across Italy. (**Panel B**): Number of beds in the hospitals where the participating centres were located. (**Panel C**): Rate of centres where the electrophysiological procedures analysed were performed. Abbreviations used in the figure. AF: atrial fibrillation; DCC: direct-current cardioversion; S-ICD: subcutaneous implantable cardioverter-defibrillator; VT: ventricular tachycardia.

A total of 105 cardiologists from 92 Italian centres took part in the survey. In 10 centres, more than one cardiologist participated in the survey (mean: 2; range: 2–4). A complete list of centres is reported in the Acknowledgments section. Of the 372 centres operating in Italy—according to 2019 AIAC census data (https://aiac.it/attivita/censimenti/, accessed on 15 April 2021)—24.7% participated in the survey. A mean of three centres per region (range: 0–13; interquartile range: 2–7) took part in the survey, with a wide geographical distribution (Figure 1, panel A). In seven out of 20 Italian regions, five or more centres participated. The response rate was similar in Northern, Central and Southern Italy (23.5, 32.6, and 21.0% of all operating centres, respectively, *p* = 0.141). 

Many participating centres (47.8%) were in hospitals with more than 400 beds, 34.8% in hospitals that had between 201 and 400 beds; and the remaining 17.4% in hospitals with 200 or fewer beds (Figure 1, panel B); 96.7% and 95.7% of the hospitals had a surgery unit and anaesthesiology-ICU, respectively.

Elective DCC of persistent atrial fibrillation (AF)/atrial flutter was performed in all participating centres. The second most widely performed procedure was S-ICD implantation (performed in 89 centres, 96.7%), followed by AF ablation (82 centres, 89.1%), and VT ablation (76 centres, 72.8%). Transvenous lead extraction was the least widespread procedure (21 centres, 22.8%) (Figure 1, panel C).

### 3.2. Elective Direct-Current Cardioversion

**Figure 2:**
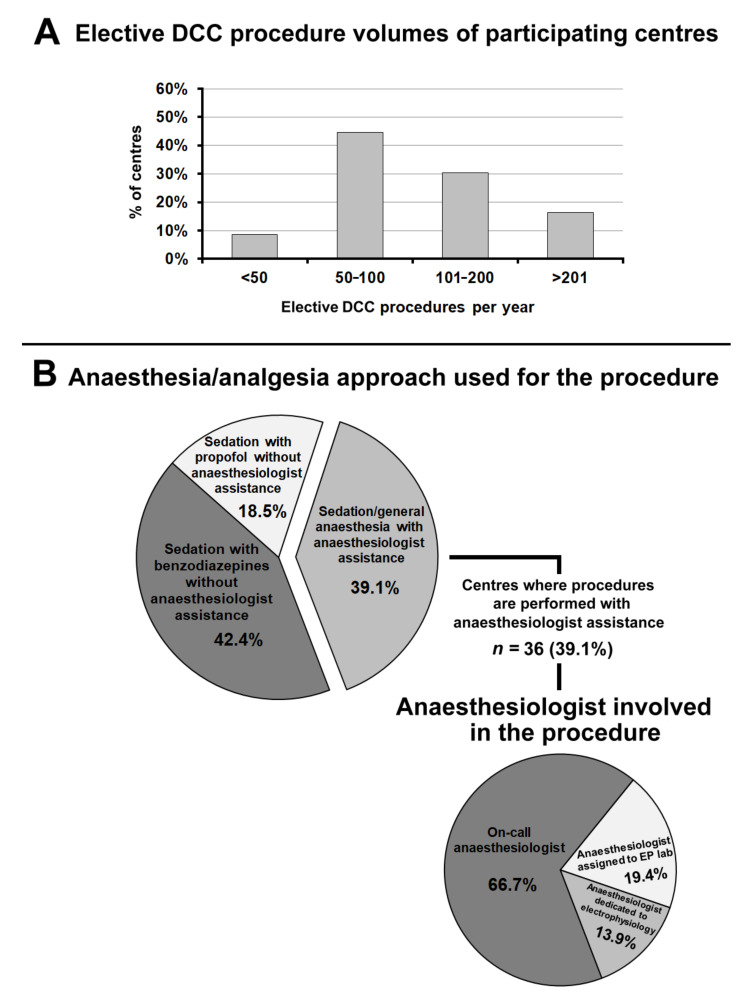
(**Panel A**): Elective direct-current cardioversion procedure volumes of participating centres. (**Panel B**): Anaesthesia/analgesia approach used for the procedure. Abbreviations used in the figure. DCC: direct-current cardioversion; EP lab: electrophysiology laboratory.

Overall, 44.6% of hospitals performed between 50 and 100 elective DCC per year, 46.7% more than 100, and only 8.7% less than 50 (Figure 2, panel A).

Figure 2, panel B summarizes the sedation/anaesthesia approach used for elective DCC. In most centres (60.9%), DCC was usually performed by a cardiologist without anaesthesiologic assistance. In two thirds of cases, the sedative used was an intravenous short-acting benzodiazepine, while in the remaining cases it was intravenous propofol. A predictor of performing DCC without anaesthesiologic assistance was an annual volume of elective DCC procedures of more than 100 per year (OR, 5.83; 95% CI, 2.25 to 15.14; *p* < 0.001). 

In 39.1% of centres, DCC was performed under deep sedation/general anaesthesia with the assistance of an anaesthesiologist. In most cases (66.7%) the anaesthesiologist involved in the procedure was the on-call anaesthesiologist, and in 19.4% of cases the anaesthesiologist was assigned to the EP lab; in 13.9% of cases (five centres) an anaesthesiologist was dedicated to electrophysiology.

### 3.3. S-ICD Implantation

**Figure 3:**
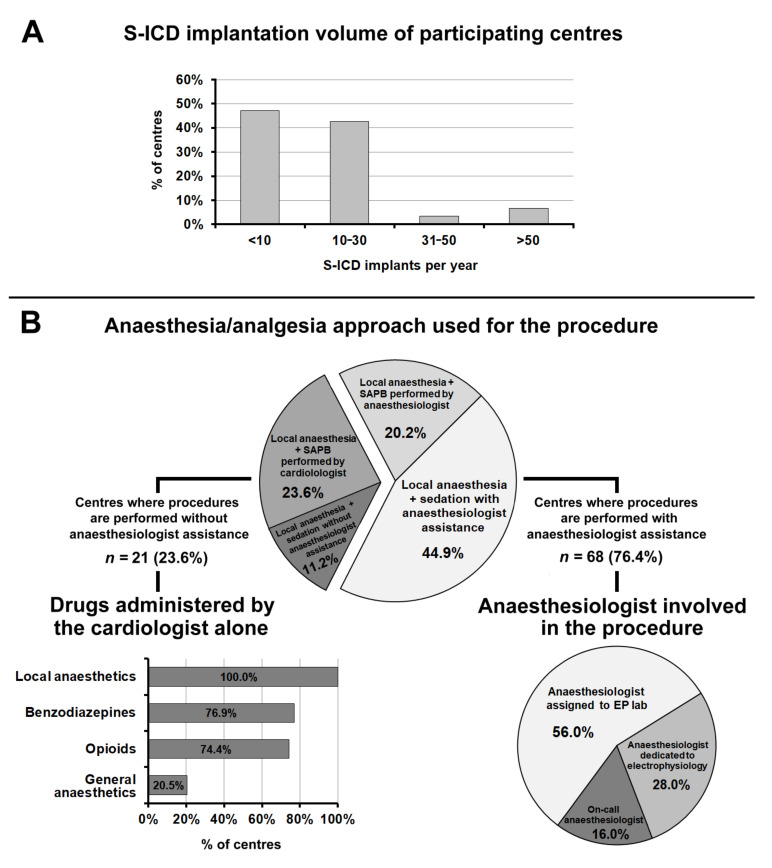
(**Panel A**): Subcutaneous implantable cardioverter-defibrillator implantation volume of participating centres. (**Panel B**): Anaesthesia/analgesia approach used for the procedure. Abbreviations used in the figure. S-ICD: subcutaneous implantable cardioverter-defibrillator; EP lab: electrophysiology laboratory.

Overall, 89 centres performed S-ICD implantations, 47.2% of which had an annual volume of less than 10 procedures per year, 42.7% from 10 to 30, and the remaining 10.1% more than 30 procedures per year (Figure 3, panel A). 

Figure 3, panel B summarizes the anaesthesia and sedation approach used for S-ICD implantation. In 23.6% of centres, the procedures were performed without an anaesthesiologist. A predictor of performing the procedure without anaesthesiologic assistance was an annual volume of S-ICD implantations of more than 30 per year (OR, 4.40; 95% CI, 1.02 to 19.03; *p* = 0.034). 

In 39 centres (43.8%) anaesthesia/analgesia was achieved by means of ultrasound-guided serratus anterior plane block (SAPB) [5]. In over half (21) of these centres, SAPB was routinely performed by cardiologists, in the remaining cases, by an anaesthesiologist. 

When S-ICD implantation was performed without anaesthesiologic assistance, the drugs (in addition to local anaesthetics) commonly administered were benzodiazepines (in 76.9% of cases), opioids (74.4%), and propofol (20.5%).

In 76.4% of centres, S-ICD implantation was performed with anaesthesiologic assistance. In most cases (56.0%) the anaesthesiologist involved in the procedure was assigned to the EP lab; in 28.0% of cases, an anaesthesiologist dedicated to electrophysiology, and in the remaining 16.0% of cases the on-call anaesthesiologist.

### 3.4. Atrial Fibrillation Ablation

**Figure 4:**
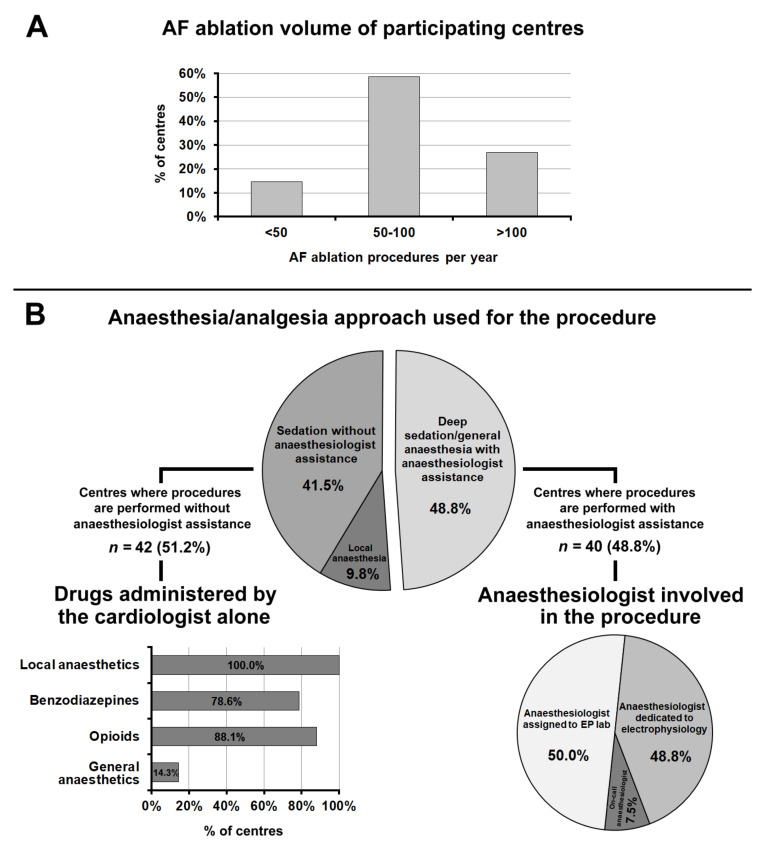
(**Panel A**): Atrial fibrillation ablation procedure volumes of participating centres. (**Panel B**): Anaesthesia/analgesia approach used for the procedure. Abbreviations used in the figure. AF: atrial fibrillation; EP lab: electrophysiology laboratory.

The majority (58.5%) of 82 participating centres performing AF ablation declared that they performed between 50 and 100 procedures per year, 26.8% more than 100, and the remaining 14.6% less than 50 (Figure 4, panel A). 

Figure 4, panel B summarizes the anaesthesia and sedation approach used for AF ablation. In 51.2% of centres, procedures were performed without anaesthesiologic assistance; in this case, the drugs most frequently administered were opioids (88.1%), benzodiazepines (78.6%), and propofol (14.3%).

In 48.8% of centres, AF ablation was performed under deep sedation/general anaesthesia with anaesthesiologic assistance. In 50.0% of cases, the anaesthesiologist involved in the procedure was assigned to the EP lab, and in 48.8% of cases, an anaesthesiologist was dedicated to electrophysiology; in the remaining 7.5% of cases, the on-call anaesthesiologist was involved.

### 3.5. Ventricular Tachycardia Ablation

**Figure 5:**
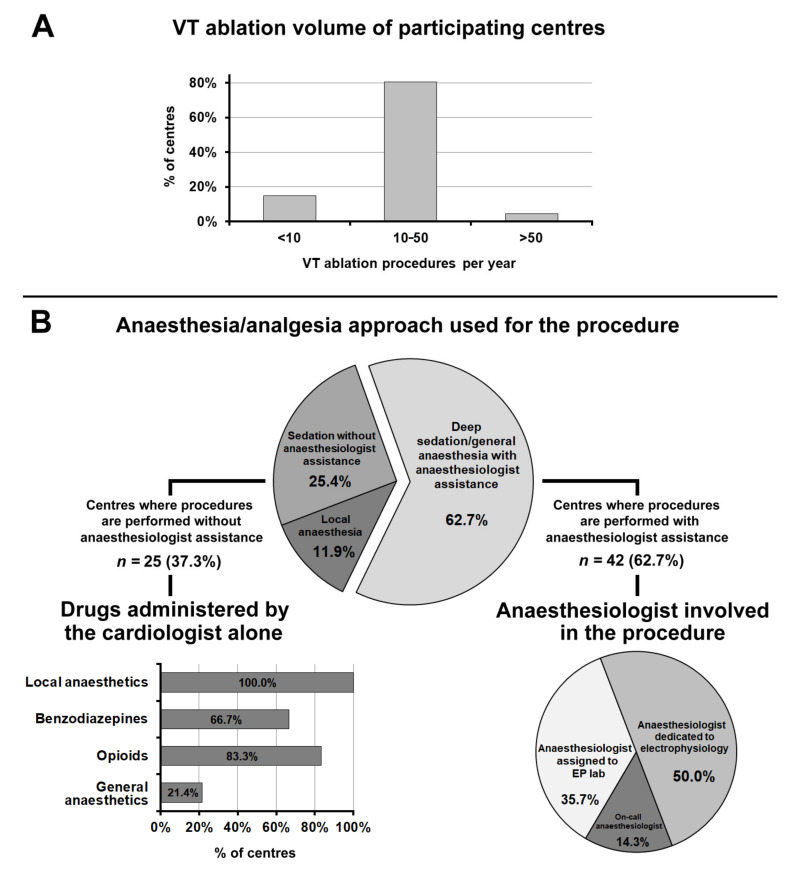
(**Panel A**): Ventricular tachycardia ablation procedure volumes of participating centres. (**Panel B**): Anaesthesia/analgesia approach used for the procedure. Abbreviations used in the figure. VT: ventricular tachycardia; EP lab: electrophysiology laboratory.

Of 67 participating centres, 80.6% performed from 10 to 50 VT ablation procedures per year, and the remaining 19.4% either less than 10 or more than 50 (Figure 5, panel A).

Figure 5, panel B summarizes the anaesthesia and sedation approach used for VT ablation; 37.3% of centres performed ablations without anaesthesiologic assistance. When the procedure was performed without the anaesthesiologist, the drugs commonly administered, in addition to local anaesthetics, were opioids (83.3%), benzodiazepines (66.7%), and propofol (21.4%).

In 62.7% of centres, VT ablation was performed under deep sedation/general anaesthesia with anaesthesiologic assistance. In 50.0% of cases, the anaesthesiologist involved in the procedure was dedicated to electrophysiology; in 35.7% of cases, an anaesthesiologist assigned to the EP lab, and in the remaining 14.3% of cases the on-call anaesthesiologist.

### 3.6. Transvenous Lead Extraction 

Of the 21 centres performing lead extraction, 18 (85.7%) performed less than 50 procedures per year, the remaining three centres more than 50.

**Figure 6:**
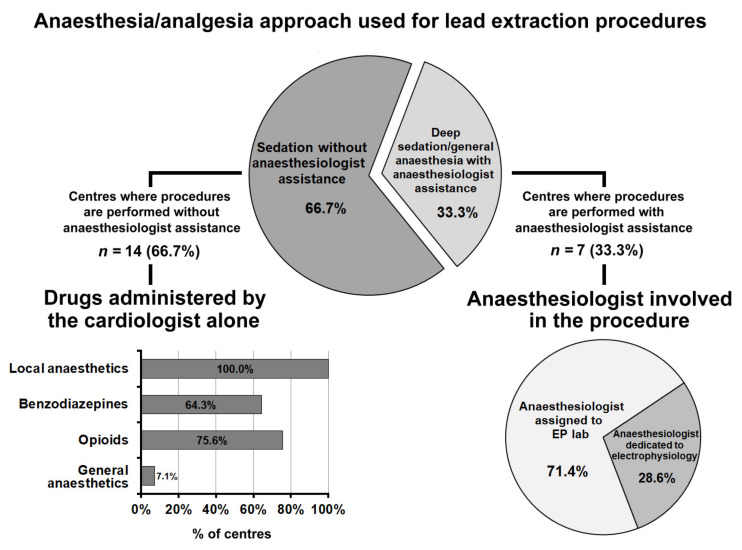
Anaesthesia/analgesia approach used for transvenous lead extraction procedures. Abbreviations used in the figure. EP lab: electrophysiology laboratory.

Figure 6 summarizes the anaesthesia and sedation approach used for transvenous lead extraction. In 66.7% of centres, lead extractions were performed without anaesthesiological assistance; in this case, the drugs most frequently administered, in addition to local anaesthetics, were opioids (75.6%), benzodiazepines (64.3%), and propofol (7.1%).

Transvenous lead extraction was performed under deep sedation/general anaesthesia with anaesthesiological assistance in seven centres (33.3%). In five of these centres, the anaesthesiologist involved in the procedure was assigned to the EP lab. In the remaining 2, the anaesthesiologist was dedicated to electrophysiology; both centres performed over 50 transvenous lead extraction procedures per year.

### 3.7. General Aspects of Sedation and Anaesthesia in Electrophysiological Procedures

In 21 centres (22.8%) all the procedures were performed without anaesthesiologic assistance, while in 13 centres (14.1%) an anaesthesiologist was involved in every procedure.

Out of 105 cardiologists, 23 (21.9%) declared that they routinely administered propofol, without the supervision of an anaesthesiologist, in at least one of the above-mentioned procedures. Operating in a high-volume ablation centre (>100 AF ablations per year, >50 VT ablations per year) was a predictor of using propofol without anaesthesiologic assistance (OR, 3.12; 95% CI, 1.13 to 8.62; *p* = 0.024).

All centres performed transvenous CIED implantation, electrophysiological study and the simple ablation procedures without anaesthesiologic assistance.

## 4. Discussion

Surveys are an efficient tool for the investigation of common clinical practice. To our knowledge, the current survey on sedation in the EP lab is the largest ever conducted on this important topic. Approximately one fourth of Italian arrhythmological centres from all over the country took part in the survey. Almost half of the procedures were performed under local sedation, owing to their low risk of complications and low level of pain [6,7,8,9]. DCC requires rapid, deep sedation; this can be achieved by means of a cardiologist-administered benzodiazepine bolus, or by anaesthesiologist-assisted sedation with propofol as both strategies seem equally safe in elective [10] and urgent settings [11]. By contrast, subcutaneous ICD implantation [5,12], AF and VT ablation [13,14] may be long and painful procedures, while lead extraction is prone to complications that could be life-threatening and may require cardiac surgery. In all these interventions, and in other emerging procedures, the anaesthesiologist may be helpful.

Benzodiazepines and opioids (in addition to local anaesthetics) are the sedatives most frequently used by cardiologists; however, a non-negligible percentage of cardiologists use general anaesthetics, such as propofol. Owing to the pharmacokinetic and pharmacodynamic characteristics of this agent, the potential sudden hemodynamic and respiratory complications associated with its use, and the lack of an antagonist (unlike in the case of other sedatives), the nonanaesthesiologist administration of propofol (NAAP) remains highly controversial and may imply medico-legal issues [15,16,17,18].

Although the available evidence suggests that cardiologist-directed use of propofol during electrophysiology procedures can be carried out without additional risk to the patient [18,19,20,21], specific recommendations from scientific associations and a specific case law are lacking.

As shown by the results of the present survey, the use of NAAP by cardiologists is not uncommon, especially in electrophysiology procedures. In order to reduce the risk of legal disputes, it is important to consider the issues of adherence to local protocols jointly defined by anaesthesiologists and cardiologists, the organization of practical training, and strict collaboration between cardiologists and anaesthesiologists, especially when patients are at higher risk.

Dexmedetomidine, an alfa-2 adrenergic agonist with a predominantly central effect, seems a valid alternative to propofol, owing to its lower respiratory depressive action and the fact that it has no, or less, effect on arrhythmia inducibility [22,23,24,25,26,27]. The use of a multi-drug scheme has proved effective and safe in atrial fibrillation ablation [28,29,30]. Furthermore, a multi-drug sedation scheme could enhance the effect of each drug, minimize adverse events, and reduce drug concentration more rapidly on suspension.

The results of this survey reveal that anaesthesiologic support is called upon in less than half of cases. Moreover, when such assistance is utilised, the anaesthesiologist is often on-call or assigned to the EP lab and is rarely a dedicated one. In a field in which technology is making great strides, the unavailability of anaesthesiologic support could be limiting and even dangerous, particularly in frail patients.

Our survey shows that the sedation and analgesia strategy currently used for interventional electrophysiology procedures in Italy is heterogeneous and depends on the type of procedure and the procedural volume of the centre. Less complex procedures, such as DCC and S-ICD implantation, are routinely performed without anaesthesiologic assistance in higher volume centres, probably because the cardiologists of these centres are more experienced in the use of sedative/anaesthetic drugs. In low-volume centres, where the involvement of the anaesthesiologist is more frequent, the anaesthesiologist is more often on-call, probably because, due to the low procedural volume of procedures, there is no availability of an anaesthesiologist to dedicate to EP lab.

In more complex procedures, such as AF and VT ablation, anaesthesiologic assistance is not routinely required in about half of the cases, and this would seem not to be influenced by the procedural volume of the centres. When these procedures are performed with anaesthesiologic assistance, the anaesthesiologist is almost always dedicated or assigned to EP lab. It is likely that, in the centres that perform complex procedures, there are structured organizational protocols that provide for the routine assignment of the anaesthesiologist to EP lab.

This survey was not influenced by the current COVID-19 pandemic, as it began before the pandemic surge, and its questions concerned pre-pandemic years. Thus, the shortage of anaesthesiologists to support cardiological procedures was already evident in the recent past. Moreover, during the pandemic, many anaesthetists have been involved in the treatment of COVID-19, further reducing their availability for elective cardiological procedures, which, consequently, have also been severely affected [31].

The wider use of regional anesthesia techniques, as in other types of surgery, could avoid the need for deep sedation during CIED implantation, thereby reducing the associated risks and costs. In this regard, the results of two recent Italian studies on S-ICD implantation showed the great value of serratus anterior plane block in terms of efficacy and safety [5,12].

Anaesthesiologic assistance is an unmet need in several centres, mostly owing to lack of staff. While shared sedation protocols might be able to compensate for this lack, their safety and efficacy should be validated in prospective studies. Gerstein NS et al. carried out a review of various sedation schemes and proposed a flow chart for sedation planning [2]. In our view, what is needed is not only a generic sedation plan but also a detailed drug infusion protocol, with a pre-specified step-up scheme, in order to gather scientific evidence in a field in which practice is driven by individual experience.

The results of this survey show that in Italian clinical practice sedation/analgesia for electrophysiology procedures is often routinely directed by the cardiologist without the supervision of an anaesthesiologist. This is especially observed in high-volume centres and for less complex procedures. Several data from the literature suggest that this strategy is safe when the cardiologist is experienced in sedative/anaesthetic drugs administration and advanced respiratory care [10,11,18,19,20,21]. In order to overcome the chronic shortage of anaesthesiologists and to standardize sedation/analgesia strategies in interventional arrhythmology procedures, it would be desirable to institute specific training programs aimed to improve the knowledge and expertise of electrophysiologists in sedative/anaesthetic drugs management.

## 5. Study Limitations

Only 92 of the 372 arrhythmia centres operating in Italy took part in the survey (24.7% of the total). For this reason, our findings should be interpreted with caution, as they may not accurately reflect Italian clinical practice.

Data on procedural details, as well patients’ characteristics and outcome were not collected, consequently, it was not possible to compare the complication rate of the procedures performed with and without anaesthesiologic assistance, as well as to know if the decision to perform a procedure with or without anaesthesiologic assistance was based on the characteristics of the patient.

Twenty of the 28 questions of the questionnaire were multiple-choice questions. This type of questionnaire is time-efficient, and responses are easy to code and interpret. On the other hand, surveys based on multiple-choice questions have some limitations, in that respondents are required to choose a response that may not exactly reflect their situation. In addition, the arbitrary design of questionnaires and multiple-choice questions with pre-conceived categories constitutes a biased and overly simple view of reality.

## 6. Conclusions

This Italian survey shows that sedation practice is inhomogeneous across the country. Differences pertain to the sedatives/general anaesthetics used and the availability of anaesthesiologic assistance. There is a lack of strong scientific evidence regarding different sedation schemes. The quality of anaesthesia is a major determinant of procedure success and safety [32]. We think that shared sedation protocols, with a detailed step-up drug scheme, are an unmet need in some electrophysiological procedures and of paramount importance for the accumulation of scientific evidence.

## Data Availability

The data presented in this study are available upon reasonable request from the corresponding author. The data are not publicly available due to privacy restrictions.

## Supplementary Materials

The following are available online at https://www.mdpi.com/article/10.3390/jcm10215035/s1, Scheme S1: Questionnaire sent to the centres.
