# Supplementary material for: The Practice of Deep Sedation in Electrophysiology and Cardiac Pacing Laboratories: Results of an Italian Survey Promoted by the AIAC (Italian Association of Arrhythmology and Cardiac Pacing)
Source: J Clin Med. 2021 Oct 28;10(21):5035. doi: 10.3390/jcm10215035 (PMC8584354; doi:10.3390/jcm10215035)
Supplement: Supplementary file 1 [file jcm-10-05035-s001.zip › jcm-1408137-supplementary.pdf]

**Supplementary material**

**Questionnaire published on the official AIAC website**

1) Number of hospital beds in your hospital: ____

2) Does your hospital have a surgical unit?
A) Yes
B) No

3) Does your hospital have an anaesthesia and intensive care unit?
A) Yes
B) No

4) Are elective direct-current cardioversions of persistent atrial fibrillation/atrial flutter performed in your centre?
A) Yes
B) No

5) Number of elective direct-current cardioversions of persistent atrial fibrillation/atrial flutter performed per year:
A) <50
B) 50-100
D) 100-200
E) >200

6) Elective direct-current cardioversions are usually performed:
A) Under sedation with benzodiazepines, without anaesthesiologist assistance
B) Under sedation with propofol, without anaesthesiologist assistance
C) Under sedation/general anaesthesia with anaesthesiologist assistance

7) If the elective direct-current cardioversions are performed with anaesthesiologist assistance, the anaesthesiologist involved in the procedure is:
A) The on-call anaesthesiologist
B) An anaesthesiologist assigned to the EP lab
C) An anaesthesiologist dedicated to electrophysiological procedures

8) Are S-ICD implantation procedures performed in your centre?
A) Yes
B) No

9) Number of S-ICD implantation procedures performed per year:
A) <10
B) 10-30
D) 31-50
E) >50

10) S-ICD implantation procedures are usually performed:
A) Under local anaesthesia

B) Under local anaesthesia + ultrasound-guided serratus anterior plane block performed by cardiologist

C) Under local anaesthesia + ultrasound-guided serratus anterior plane block performed by anaesthesiologist

D) Under local anaesthesia + sedation without anaesthesiologist assistance

E) Under local anaesthesia + sedation with anaesthesiologist assistance

11) If the S-ICD implantation procedures are performed with anaesthesiologist assistance, the anaesthesiologist involved in the procedure is:

A) The on-call anaesthesiologist
B) An anaesthesiologist assigned to the EP lab
C) An anaesthesiologist dedicated to electrophysiological procedures

12) If the S-ICD implantation procedures are performed without anaesthesiologist assistance, the drugs administered by the cardiologist alone are (multiple answers are allowed):

A) Local anaesthetics
B) Benzodiazepines
C) Opioids (such as fentanyl, remifentanil, morphine)
D) General anaesthetics (such as propofol)

13) Are AF ablation procedures performed in your centre?

A) Yes
B) No

14) Number of AF ablation procedures performed per year:

A) <50
B) 50-100
C) 101-300
D) >300

15) AF ablation procedures are usually performed:

A) Under local anaesthesia
B) Under sedation without anaesthesiologist assistance
E) Under deep sedation/general anaesthesia with anaesthesiologist assistance

16) If the AF ablation procedures are performed with anaesthesiologist assistance, the anaesthesiologist involved in the procedure is:

A) The on-call anaesthesiologist
B) An anaesthesiologist assigned to the EP lab
C) An anaesthesiologist dedicated to electrophysiological procedures

17) If the AF ablation procedures are performed without anaesthesiologist assistance, the drugs administered by the cardiologist alone are (multiple answers are allowed):

A) Local anaesthetics
B) Benzodiazepines
C) Opioids (such as fentanyl, remifentanil, morphine)
D) General anaesthetics (such as propofol)

18) Are VT ablation procedures performed in your centre?

A) Yes
B) No

19) Number of VT ablation procedures performed per year:
- A) <10
- B) 10-50
- C) 51-100
- D) >100

20) VT ablation procedures are usually performed:
- A) Under local anaesthesia
- B) Under sedation without anaesthesiologist assistance
- E) Under deep sedation/general anaesthesia with anaesthesiologist assistance

21) If the VT ablation procedures are performed with anaesthesiologist assistance, the anaesthesiologist involved in the procedure is:
- A) The on-call anaesthesiologist
- B) An anaesthesiologist assigned to the EP lab
- C) An anaesthesiologist dedicated to electrophysiological procedures

22) If the VT ablation procedures are performed without anaesthesiologist assistance, the drugs administered by the cardiologist alone are (multiple answers are allowed):
- A) Local anaesthetics
- B) Benzodiazepines
- C) Opioids (such as fentanyl, remifentanil, morphine)
- D) General anaesthetics (such as propofol)

23) Are lead extraction procedures performed in your centre?
- A) Yes
- B) No

24) Number of lead extraction procedures performed per year:
- A) <10
- B) 10-50
- C) 51-100
- D) >100

25) Lead extraction procedures are usually performed:
- A) Under local anaesthesia
- B) Under sedation without anaesthesiologist assistance
- E) Under deep sedation/general anaesthesia with anaesthesiologist assistance

26) If the lead extraction procedures are performed with anaesthesiologist assistance, the anaesthesiologist involved in the procedure is:
- A) The on-call anaesthesiologist
- B) An anaesthesiologist assigned to the EP lab
- C) An anaesthesiologist dedicated to electrophysiological procedures

27) If the lead extraction procedures are performed without anaesthesiologist assistance, the drugs administered by the cardiologist alone are (multiple answers are allowed):
- A) Local anaesthetics
- B) Benzodiazepines
- C) Opioids (such as fentanyl, remifentanil, morphine)

D) General anaesthetics (such as propofol)

28) In your centre, anaesthesiologist assistance is usually required for patient sedation in the following procedures (multiple answers are allowed):

A) Simple pacing procedures (implantation of pacemaker, single- or dual-chamber ICD, excluding S-ICD)

B) Complex pacing procedures (implantation of biventricular devices, upgrading, leadless pacemakers, excluding S-ICD)

C) Endocavitary electrophysiological studies

D) Simpler ablation procedures

E) None of the above

List of abbreviations

AF: atrial fibrillation
EP lab: electrophysiology laboratory
ICD: implantable cardioverter-defibrillator
S-ICD: subcutaneous implantable cardioverter-defibrillator
VT: ventricular tachycardia
